# Upregulation of neuronal astrocyte elevated gene-1 protects nigral dopaminergic neurons *in vivo*

**DOI:** 10.1038/s41419-018-0491-3

**Published:** 2018-04-18

**Authors:** Eunju Leem, Hyung-Jun Kim, Minji Choi, Sehwan Kim, Yong-Seok Oh, Kea Joo Lee, Young-Shik Choe, Jae-Young Um, Won-Ho Shin, Jae Yeong Jeong, Byung Kwan Jin, Dong Woon Kim, Catriona McLean, Paul B. Fisher, Nikolai Kholodilov, Kwang Seok Ahn, Jae Man Lee, Un Ju Jung, Seok-Geun Lee, Sang Ryong Kim

**Affiliations:** 1 School of Life Sciences, BK21 Plus KNU Creative BioResearch Group, Institute of Life Science & Biotechnology, Kyungpook National University, Daegu, 41566 Republic of Korea; 2 Department of Neural Development and Disease, Department of Structure & Function of Neural Network, Korea Brain Research Institute, Daegu, 41062 Republic of Korea; 3 Department of Science in Korean Medicine, Graduate School, Kyung Hee University, Seoul, 02447 Republic of Korea; 4 Department of Brain-Cognitive Science, Daegu-Gyeongbuk Institute of Science and Technology, Daegu, 42988 Republic of Korea; 5 Predictive Model Research Center, Korea Institute of Toxicology, Korea Research Institute of Chemical Technology, Daejeon, 34114 Republic of Korea; 6 Department of Biochemisry and Molecular Biology, Department of Neuroscience Graduate School, School of Medicine, Kyung Hee University, Seoul, 02447 Republic of Korea; 7 Department of Anatomy, Brain Research Institute, School of Medicine, Chungnam National University, Daejeon, 34134 Republic of Korea; 8 Victorian Brain Bank Network, Florey Institute of Neuroscience and Mental Health, The University of Melbourne, Melbourne, VIC 3004 Australia; 9 Department of Anatomical Pathology, Alfred Hospital, Melbourne, VIC 3004 Australia; 10 Department of Human and Molecular Genetics, VCU Institute of Molecular Medicine, VCU Massey Cancer Center, School of Medicine, Virginia Commonwealth University, Richmond, VA 23298 USA; 11 Department of Neurology, Columbia University, New York, NY 10032 USA; 12 Department of Biochemistry and Cell Biology, Cell and Matrix Research Institute, BK21 Plus KNU Biomedical Convergence Program, School of Medicine, Kyungpook National University, Daegu, 41944 Republic of Korea; 13 Department of Food Science and Nutrition, Pukyong National University, Busan, 48513 Republic of Korea; 14 KHU-KIST Department of Converging Science and Technology, Kyung Hee University, Seoul, 02447 Republic of Korea; 15 Brain Science and Engineering Institute, Kyungpook National University, Daegu, 41944 Republic of Korea

## Abstract

The role of astrocyte elevated gene-1 (AEG-1) in nigral dopaminergic (DA) neurons has not been studied. Here we report that the expression of AEG-1 was significantly lower in DA neurons in the postmortem substantia nigra of patients with Parkinson’s disease (PD) compared to age-matched controls. Similarly, decreased AEG-1 levels were found in the 6-hydroxydopamine (6-OHDA) mouse model of PD. An adeno-associated virus-induced increase in the expression of AEG-1 attenuated the 6-OHDA-triggered apoptotic death of nigral DA neurons. Moreover, the neuroprotection conferred by the AEG-1 upregulation significantly intensified the neurorestorative effects of the constitutively active ras homolog enriched in the brain [Rheb(S16H)]. Collectively, these results demonstrated that the sustained level of AEG-1 as an important anti-apoptotic factor in nigral DA neurons might potentiate the therapeutic effects of treatments, such as Rheb(S16H) administration, on the degeneration of the DA pathway that characterizes PD.

## Introduction

Astrocyte elevated gene-1 (AEG-1), also known as metadherin, was originally identified as a human immunodeficiency virus-1- and tumor necrosis factor-alpha-inducible gene in human fetal astrocytes, and its upregulation is a well-established important oncogenic event in various types of human cancer^1–4^. The downregulation of neuronal AEG-1 has recently been shown to reduce the viability of motor neurons in a mouse model of amyotrophic lateral sclerosis (ALS) by activating apoptotic signaling pathways *via* inhibition of the phosphatidylinositol-4,5-bisphosphate 3-kinase/protein kinase B (PI3K/Akt) signaling pathway^5^.

The aberrant activation of apoptotic signaling pathways in the adult brain is a well-known neurotoxic event that is associated with neuronal loss, such as that observed in neurodegenerative diseases, including Parkinson’s disease (PD) and Alzheimer’s disease (AD)^6–8^, and the PI3K/Akt/mammalian target of rapamycin complex 1 (mTORC1) signaling pathway has been shown to elicit neuroprotective effects on the survival and growth of neurons in the nigrostriatal dopaminergic (DA) system^9–11^. However, little is known about the neuroprotective role of AEG-1 in PD.

Here we found that the loss of DA neurons in postmortem substantia nigra (SN) tissue from patients with PD were associated with significant decreases in the levels of expression of AEG-1 in nigral DA neurons of patients with PD compared to age-matched controls. These findings suggested that the relationship between AEG-1 downregulation and the pathogenesis of PD are clinically relevant. To investigate the role of AEG-1 as a survival factor in nigral DA neurons in the adult brain, we examined the effects of the adeno-associated virus (AAV)-mediated overexpression of AEG-1 on these neurons in the 6-hydroxydopamine (6-OHDA)-treated animal model of PD^9,10,12^. Additionally, we examined whether the neuroprotection conferred by AEG-1 overexpression, which might be a therapeutic intervention, contributed to the neurorestorative effects on the *in vivo* nigrostriatal DA system of treatment strategies, such as the administration of constitutively active ras homolog enriched in brain (with a S16H mutation) [Rheb(S16H)], which induces axonal regrowth in damaged DA neurons^9,10^.

## Results

### Decreased levels of AEG-1 expression in the SN of patients with PD and a neurotoxin-based model of PD

**Fig. 1:**
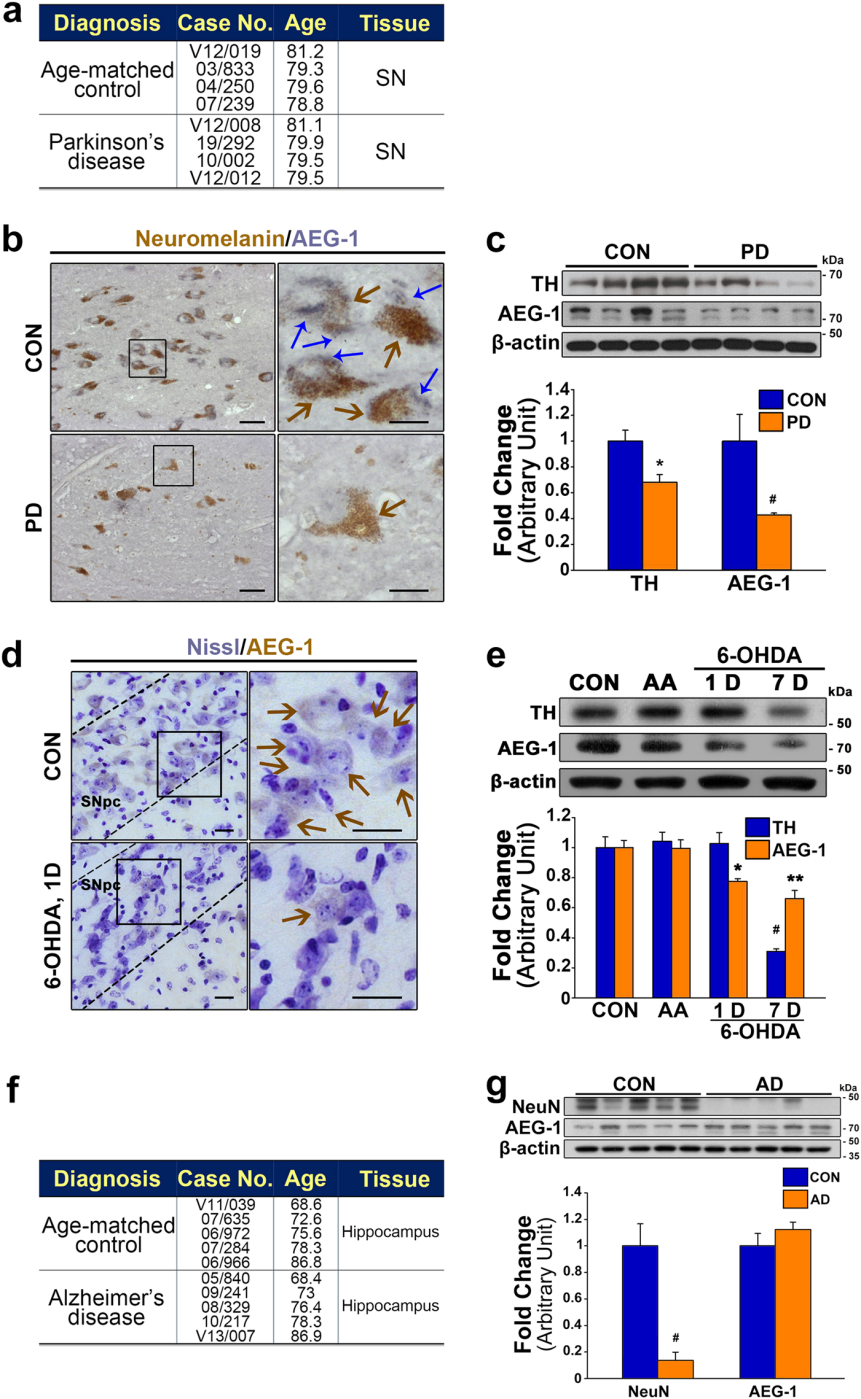
Decreased levels of astrocyte elevated gene-1 (AEG-1) in the postmortem substantia nigra (SN) of patients with Parkinson’s disease (PD) and the SN of 6-hydroxydopamine (6-OHDA)-treated mice. **a** Description of the human postmortem SN tissue. **b** Immunohistochemistry for AEG-1 in the human SN. Scale bar, 50 μm. The square insets in the left panels contain magnifications of the photomicrographs in the right panels. Scale bar, 20 μm. The blue arrows indicate AEG-1 immunoreactivity, and the brown arrows indicate neuromelanin immunoreactivity. **c** Western blot analysis of the levels of tyrosine hydroxylase (TH) and AEG-1 in the human SN. *p = 0.022 and ^#^*p* = 0.033 *vs*. age-matched controls (CON) (*t*-test; *n* = 4 for each group). **d** Representative sections showing AEG-1 expression (with Nissl counterstaining) in the mouse SN pars compacta (SNpc), which is outlined by the dotted lines. Scale bar, 20 μm. The square insets in the left contain magnifications of the photomicrographs in the right panels. Scale bar, 20 μm. **e** Western blot analyses of the levels of AEG-1 and TH in the mouse SN. AA ascorbic acid. *p = 0.024 and **p = 0.001 *vs*. intact CON; ^#^*p* < 0.001 for TH, significantly different from CON [one-way analysis of variance (ANOVA) with Tukey’s *post hoc* test; *n* = 4 for each group]. **f** Description of the human postmortem hippocampal tissue. **g** Western blot analyses of the levels of AEG-1 and neuronal nuclei (NeuN) in the hippocampus of patients with Alzheimer’s disease (AD) and CON. Please note that of the levels of AEG-1 are not decreased in the postmortem hippocampus of patients with AD compared with CON. ^#^*p* = 0.001 *vs*. CON (*t*-test; *n* = 5 for each group)

To investigate the alterations in the levels of AEG-1 expression in the SN of patients with PD (Fig. 1a), we performed immunohistochemical staining of the expression patterns (Fig. 1b) and quantified the changes using western blotting (Fig. 1c). AEG-1-positive immunoreactivity (blue) was clearly reduced in neuromelanin-positive DA neurons (brown) in the SN of patients with PD compared to age-matched controls (Fig. 1b). Western blot analyses revealed significant decreases in the levels of AEG-1 and tyrosine hydroxylase (TH, a marker of DA neurons) in the SN of the patients with PD compared to age-matched controls (Fig. 1c; ^#^*p* = 0.033 and *p = 0.022 for AEG-1 and TH, respectively, *vs*. CON). However, decreased AEG-1 expression was not observed in the hippocampus of patients with AD compared to age-matched controls, even though there was a significant loss of neuronal nuclei (NeuN, a marker of neurons) in that region in the patients compared to controls (Fig. 1f, g; ^#^*p* = 0.001 *vs*. CON). The reduction of AEG-1 (brown) was specific to the SN pars compacta of 1 day post-lesion 6-OHDA-treated mice (Fig. 1d), which is a well-known neurotoxin-based model of PD^9,10,12–14^. Western blot analyses similarly showed a significant decrease in the levels of AEG-1 expression in the SN after 6-OHDA administration, compared to untreated controls, 1 day post-lesion (Fig. 1e; *p = 0.024 *vs*. CON), even though the levels of TH were not significantly decreased in the SN (Fig. 1e).

### Decreased levels of apoptotic signaling molecules by AEG-1 transduction of mature neurons in the SN

**Fig. 2:**
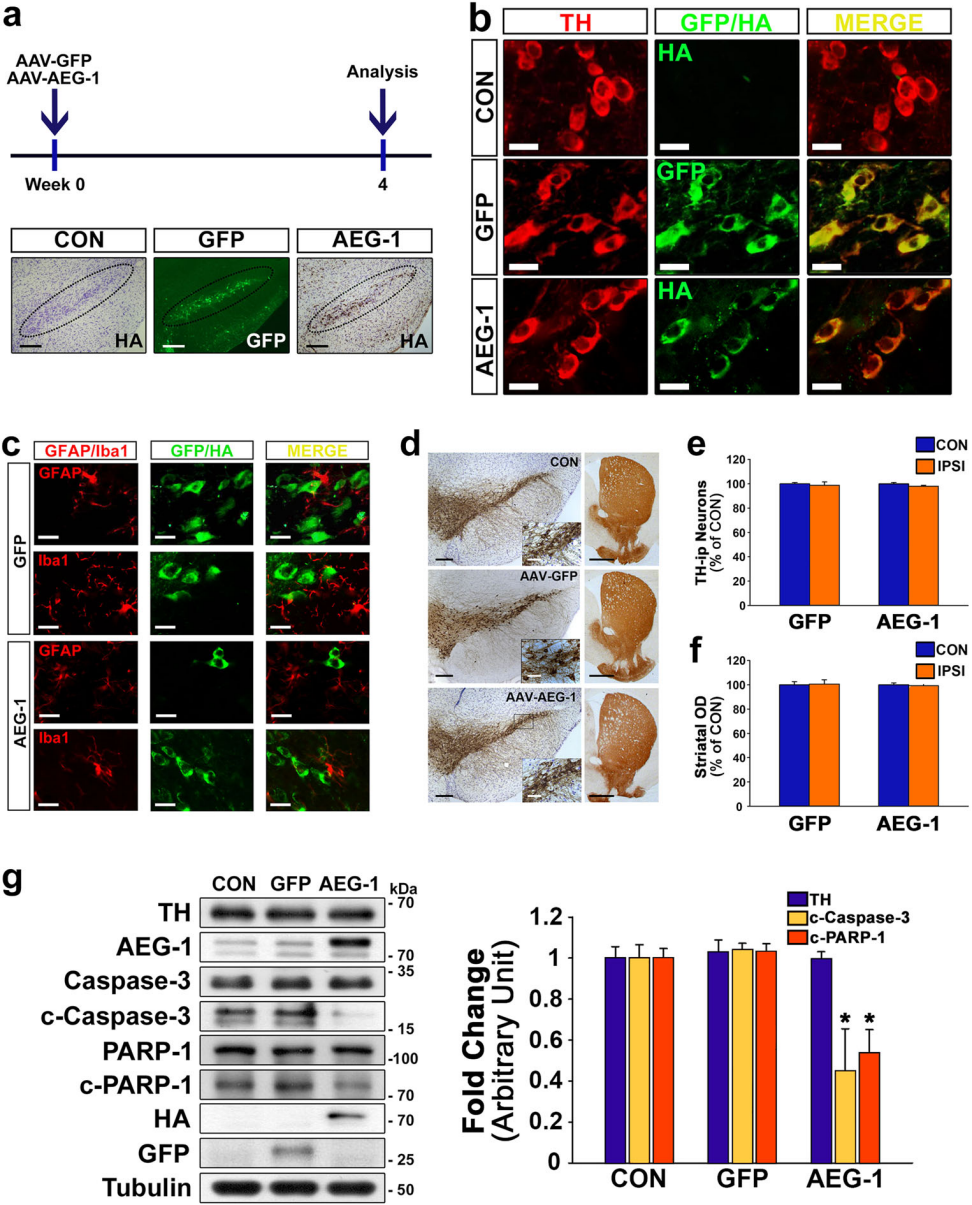
Adeno-associated virus (AAV)-AEG-1 transduction of dopaminergic (DA) neurons in the *in vivo* SN of healthy mice. **a** Experimental schematic and the immunostaining for green fluorescent protein (GFP; green) and hemagglutinin (HA; brown) in the SNpc, which is outlined by the dotted elliptical shape, which was conducted following each viral injection. Scale bar, 200 μm. **b** Representative double immunofluorescent labeling of TH (red) and GFP (green) or TH and HA (green) in the SNpc. Scale bar, 20 μm. **c** Representative double immunofluorescent labeling for glial fibrillary acidic protein (GFAP)/ionized calcium binding adaptor molecule 1 (Iba1; red), which are markers of astrocytes and microglia, respectively, and GFP/HA (green) in the SNpc of healthy mice. Scale bar, 20 μm. **d** Immunostaining for TH in the SN and striatum (STR). Scale bars, 200 μm (black) and 50 μm (white) for the SN, and 1000 μm for the STR. **e**, **f** The number and optical density of the nigral TH-positive neurons and striatal TH-positive fibers, respectively (one-way ANOVA with Tukey’s *post hoc* test; *n* = 4 for each group). CON contralateral side, IPSI ipsilateral side. **g** Western blot analyses of the levels of cleaved caspase-3 (c-caspase-3) and cleaved poly (ADP-ribose) polymerase 1 (c-PARP-1) following AEG-1 transduction in the SN of healthy brains. *p = 0.005 *vs*. CON (one-way ANOVA with Tukey’s *post hoc* test; *n* = 4 for each group)

To determine whether the upregulation of AEG-1 affected the apoptotic signaling pathways in nigral DA neurons, we evaluated the *in vivo* effects of AEG-1 overexpression on the basal levels of apoptotic markers, such as cleaved caspase-3 and cleaved poly (ADP-ribose) polymerase 1 (PARP-1) in nigral DA neurons. Mice were sacrificed 4 weeks after intranigral injections of AAV-AEG-1 or the control vector AAV-green fluorescent protein (GFP), and the transduction of DA neurons was confirmed by the patterns of GFP expression and the immunoperoxidase staining of the hemagglutinin (HA) epitope in the AAV-AEG-1 vector, respectively (Fig. 2a). HA- and GFP-positive cells were clearly colocalized with TH-positive DA neurons (Fig. 2b) but not with glial fibrillary acidic protein (GFAP)-positive astrocytes or ionized calcium binding adaptor molecule 1 (Iba1)-positive microglia in the SN (Fig. 2c). Upregulation of AEG-1, which showed no neurotoxicity (Fig. 2d*–*f), resulted in a significant decrease in the basal levels of cleaved caspase-3 and cleaved PARP-1 in the SN compared to noninjected and GFP controls (Fig. 2g; *p = 0.005 *vs*. CON).

**Fig. 3:**
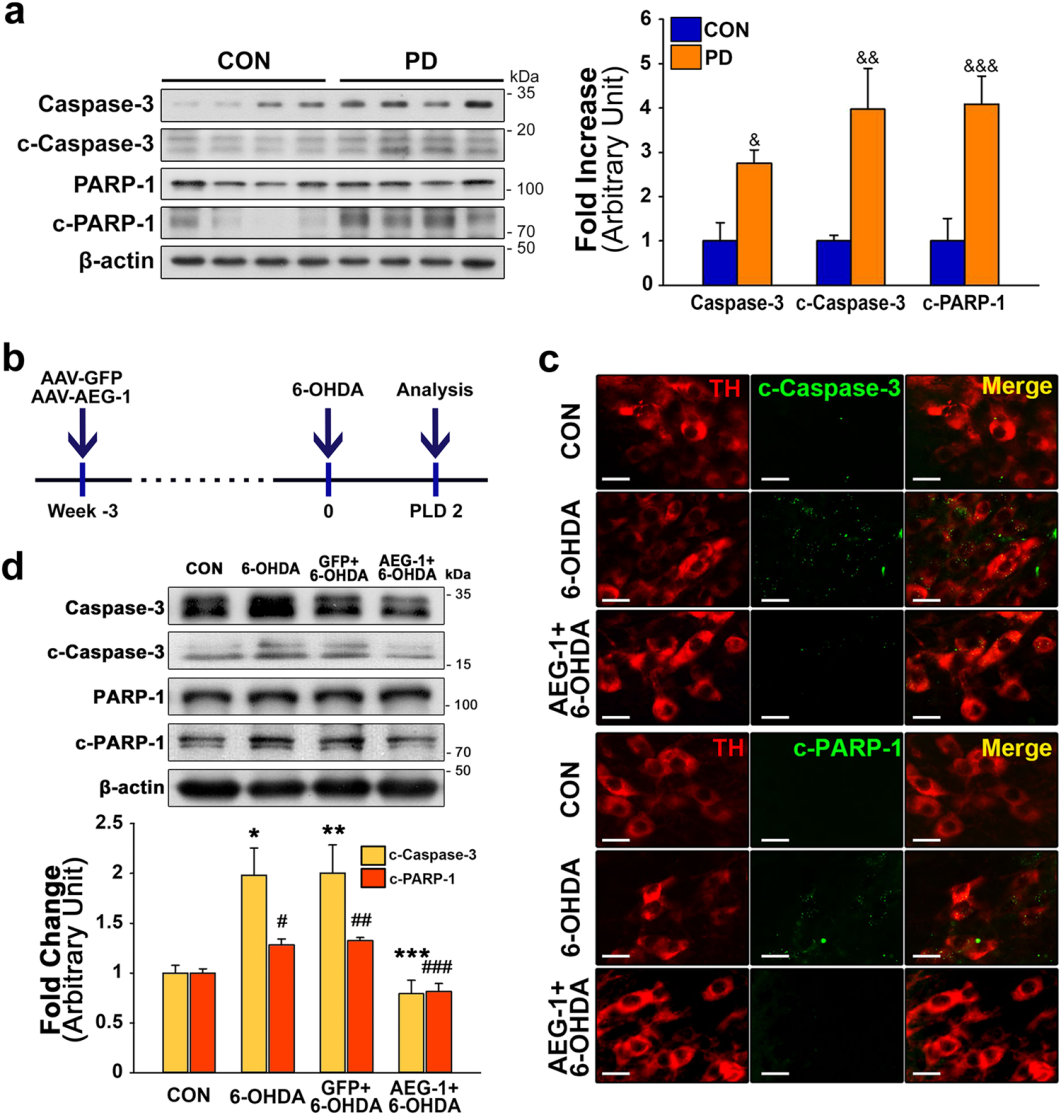
Anti-apoptotic effects of AEG-1 transduction in DA neurons on 6-OHDA neurotoxicity. **a** Western blot analyses show a significant increase in the levels of caspase-3, c-caspase-3, and c-PARP1 in the postmortem tissues of patients with PD compared with CON. ^&^*p* = 0.014 for caspase-3, ^&&^*p* = 0.019 for c-caspase-3, and ^&&&^*p* = 0.009 for c-PARP-1, *vs*. CON (*t*-test; *n* = 4 for each group). **b** Experimental schematic for Fig. 3c, d. **c** Representative double immunofluorescence labeling for TH (red) and c-caspase-3 (green) or TH and c-PARP-1 (green) in the mouse SN. AEG-1 upregulation induces reductions in the levels of expression of both c-caspase-3 and c-PARP-1 in TH-positive DA neurons in the SN with 6-OHDA neurotoxicity. Scale bar, 20 μm. **d** Western blot analyses of the levels of c-caspase-3 and c-PARP-1 in the SN 2 days after 6-OHDA treatment. *p = 0.029, **p = 0.025, ^#^*p* = 0.032, and ^##^*p* = 0.016 *vs*. CON; ***p = 0.009 and ^###^*p* = 0.002 *vs*. 6-OHDA alone (one-way ANOVA with Tukey’s *post hoc* test; *n* = 4 for each group)

Similar to previous reports that have implicated apoptosis in the loss of DA neurons in patients with PD^6,8^, western blot analyses revealed significant increases in the levels of caspase-3, cleaved caspase-3, and cleaved PARP-1 were observed in the SN of patients with PD compared to age-matched controls (Fig. 3a; ^&^*p* = 0.014, ^&&^*p* = 0.019, and ^&&&^*p* = 0.009 *vs*. CON, respectively). As shown in the experimental schematic (Fig. 3b), the double immunofluorescence staining of TH (red) and cleaved caspase-3 (green) and of TH and cleaved PARP-1 (green) (Fig. 3c), and western blot analyses (Fig. 3d) showed that the levels of both cleaved caspase-3 and cleaved PARP-1 significantly increased 2 days post-lesion in the TH-positive DA neurons in the SN of mice injected with 6-OHDA only^15,16^. However, the upregulation of AEG-1 significantly inhibited the cleavage of both caspase-3 and PARP-1 in the nigral DA neurons following the 6-OHDA injections compared to injections of 6-OHDA alone (Fig. 3c, d; ***p = 0.009 and ^###^*p* = 0.002, respectively, *vs*. 6-OHDA alone). The anti-apoptotic effects of AEG-1 on the 6-OHDA-induced neurotoxicity in DA neurons were confirmed with western blot analyses of the B-cell lymphoma 2/Bcl-2-associated X protein (Bcl-2/Bax) ratio (Supplementary Figure S1).

### Neuroprotective effects of AEG-1 upregulation against 6-OHDA neurotoxicity

**Fig. 4:**
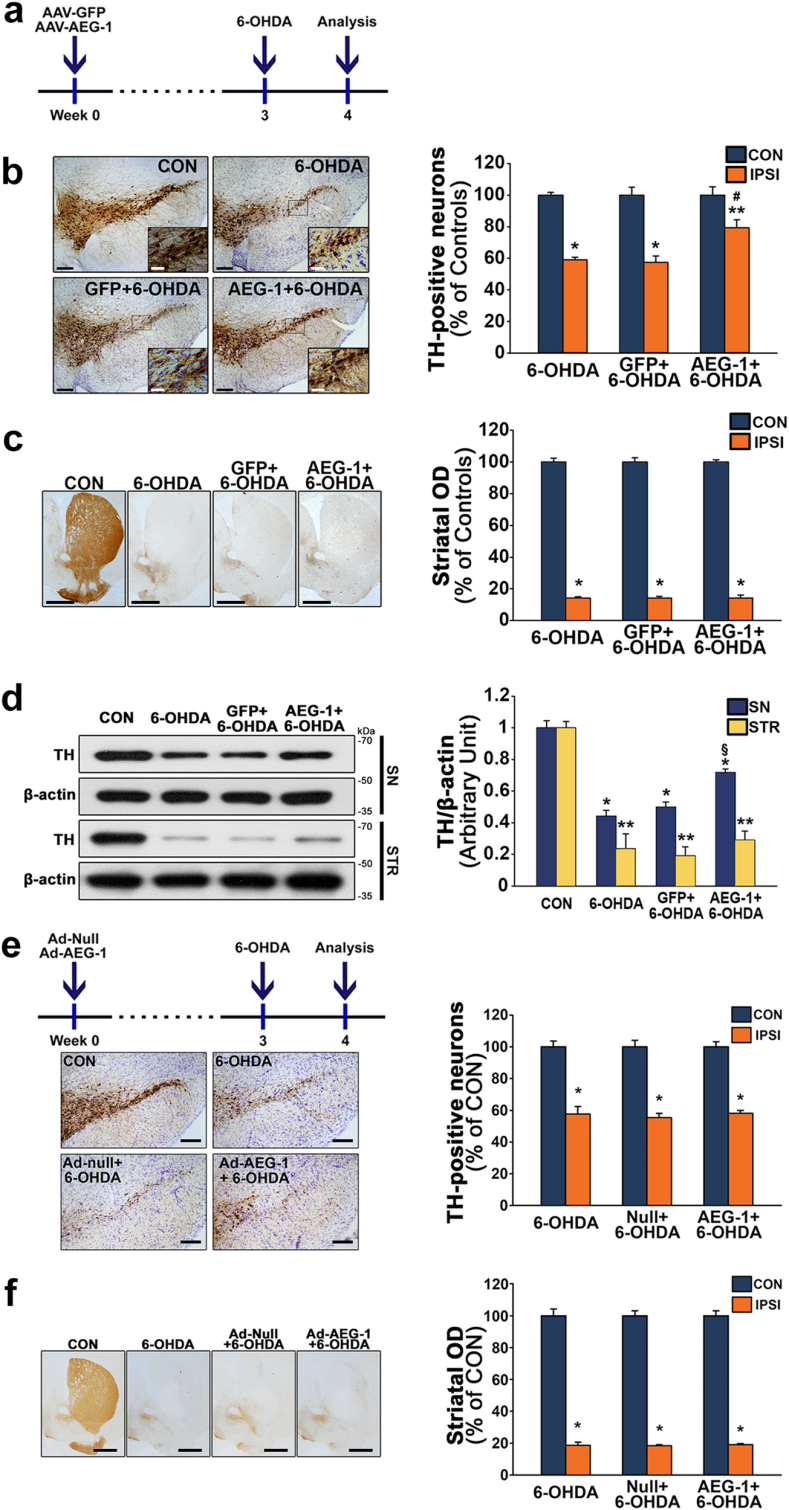
Upregulation of neuronal AEG-1 protects DA neurons from 6-OHDA-induced neurotoxicity. **a** Experimental schematic for **b**–**d**. **b** Representative coronal sections of the SN stained with anti-TH at 7 days post-lesion. Representative high-power micrographs are shown in the inset to aid visualization. Scale bars, 200 μm (black) and 50 μm (white). The quantitative analysis shows a population of preserved TH-positive neurons in the SN. *p < 0.001 and **p = 0.023 *vs*. CON; ^#^*p* = 0.026 *vs*. 6-OHDA (*n* = 4 for each group). **c** Neuroprotective effects of AEG-1 are not observed on striatal TH-positive fibers. Scale bar, 1000 μm. The histogram shows the optical densities of the striatal TH-positive fibers. *p < 0.001 *vs*. CON (*n* = 4 for each group). **d** Western blot analysis of the levels of TH with 6-OHDA-induced neurotoxicity in the nigrostriatal DA system. *p < 0.001 for SN and **p < 0.001 for STR *vs*. CON; ^§^*p* = 0.002 *vs*. 6-OHDA alone (*n* = 4 for each group). **e**, **f** Experimental schematic and representative coronal sections show that glial AEG-1 upregulation by Ad transduction does not protect the nigrostriatal DA system in the 6-OHDA-treated mouse model of PD. Scale bars, 200 μm for SN and 1000 μm for STR. *p < 0.001 *vs*. CON (*n* = 4 for each group). One-way ANOVA with Tukey’s *post hoc* test was used in **b**–**f**

We evaluated the neuroprotective effects of AAV-AEG-1 in the 6-OHDA-treated mouse model of PD (Fig. 4a). 6-OHDA administration clearly caused neurotoxicity in the nigrostriatal DA projections (Fig. 4b, c), and the transduction of DA neurons with AEG-1 but not GFP effectively mitigated the 6-OHDA-induced neurotoxicity in the SN compared to the effects of treatment with 6-OHDA alone (Fig. 4b; ^#^*p* = 0.026 *vs*. 6-OHDA alone). Western blot analyses also showed that AEG-1 transduction significantly preserved the levels of TH expression following 6-OHDA-induced neurotoxicity compared to treatment with 6-OHDA alone (Fig. 4d; ^§^*p* = 0.002 *vs*. 6-OHDA alone) in the SN but not in the striatum (STR). Similar to the limited neuroprotective effects observed using immunostaining and western blotting, the results obtained with reversed-phase high-performance liquid chromatography (HPLC) analyses indicated that the levels of striatal dopamine and its metabolites, including 3,4-dihydroxyphenylacetic acid (DOPAC) and homovanillic acid (HVA), did not significantly differ between the mice treated with 6-OHDA following AEG-1 transduction and those treated with 6-OHDA alone (Supplementary Figure S2).

To evaluate the effects of glial AEG-1 in the nigrostriatal DA system, we unilaterally injected adenovirus (Ad)-AEG-1 (Ad-AEG-1) or Ad-null, which was a control vector, in the SN of healthy mice and examined whether any neuroprotective effects were observed against 6-OHDA neurotoxicity. Four weeks after intranigral injections of Ad-AEG-1, immunohistochemical staining of the HA epitope tag for Ad-AEG-1 indicated the site-specific transduction of SN microglia with Ad-AEG-1 (Supplementary Figures S3a to c), and no neurotoxicity was observed in the nigrostriatal DA projections in the brains of the healthy mice (Supplementary Figures S3d and e). However, we did not observe neuroprotective effects of the overexpression of microglial AEG-1 against 6-OHDA-induced neurotoxicity at 1 week post-lesion (Fig. 4e, f).

### Lack of activation of the Akt/mTORC1 signaling pathway in nigral DA neurons by AEG-1 upregulation

The results of previous studies suggested that the Akt/mTORC1 signaling pathway could be regulated by changes in AEG-1 expression^3–5,17,18^, and the activation of Akt/mTORC1 signaling pathway could regulate the autophagy–lysosomal pathway (ALP), that is associated with axonal degeneration in PD^12,19^. Moreover, the sustained activation of the Akt/mTORC1 signaling pathway induces axonal regeneration in damaged neurons^9,10,20,21^. However, western blot analyses showed that the overexpression of AEG-1 alone did not alter the phosphorylation statuses of the Thr37/46 residues of 4E-BP1, which are indicative of mTORC1 activity (Supplementary Figure S4). Similarly, AEG-1 overexpression did not induce significant increases in the levels of p-Akt, which is associated with activation of mTORC1, compared to the levels in noninjected controls (Supplementary Figure S4). Additionally, no significant changes were observed in the levels of microtubule-associated protein 1A/1B-light chain 3 (LC3)-I and II, which are used as an indicator of autophagosome formation^12,19,22^, following injections of AAV-AEG-1 in the SN of healthy mice compared to those in noninjected controls (Supplementary Figure S4).

In the SN of patients with PD, we observed significant increases in the levels of LC3-II and p62, which are well-known markers of ALP^12,19,22,23^, compared to those in age-matched controls (Supplementary Figure S5a). Consistent with the increase in the levels of LC3-II and p62, a significant decrease in the levels of p-4E-BP1 expression was also observed in the SN of patients with PD (Supplementary Figure S5a). These observations suggested that the suppression of aberrant ALP through the activation of the mTORC1 signaling pathway might also be associated with neuroprotection of the nigrostriatal DA system. However, AEG-1 overexpression in DA neurons did not suppress the aberrant accumulation of autophagic components, such as LC3-II and p62, and the decrease in mTORC1 activity following 6-OHDA neurotoxicity (Supplementary Figure S5b).

### Application of AEG-1-induced neuroprotection to the functional recovery of the disrupted nigrostriatal DA system

**Fig. 5:**
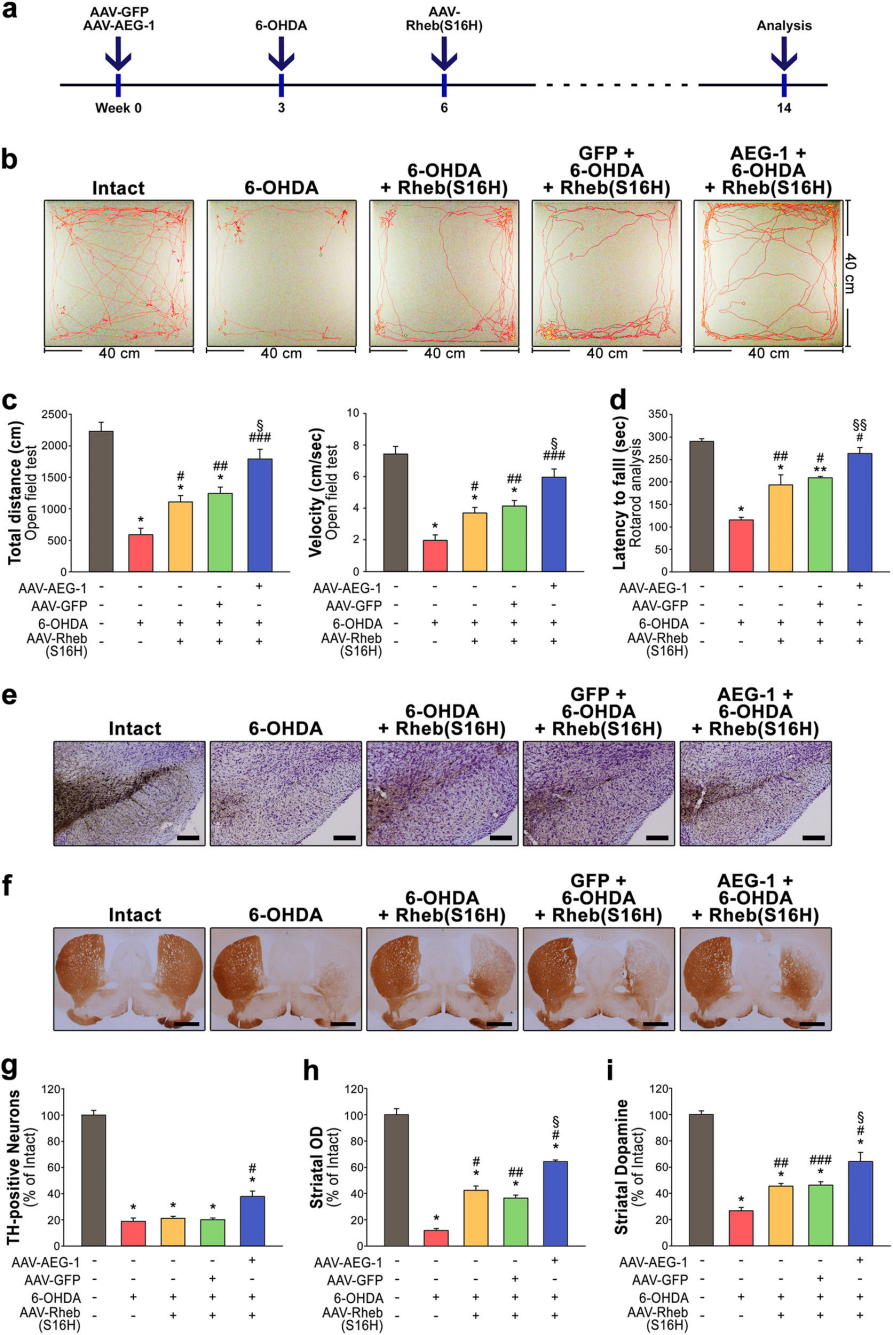
Synergistic effects of AEG-1 and Rheb(S16H) in the disrupted nigrostriatal DA system *in vivo*. **a** Experimental schematic. **b**, **c** Total distance traveled for 5 min and velocity in the open-field test. *p < 0.001 *vs*. intact controls; ^##^*p* = 0.010 and ^###^*p* < 0.001 *vs*. 6-OHDA alone; ^§^*p* = 0.007 *vs*. 6-OHDA + AAV-Rheb(S16H) group (one-way ANOVA with Tukey’s *post hoc* test; *n* = 5 for each group). ^#^*p* = 0.008 *vs*. 6-OHDA alone (*t*-test). **d** Motor deficits measured by using the rotarod test. *p < 0.001 and **p = 0.002 *vs*. intact controls; ^#^*p* < 0.001 and ^##^*p* = 0.002 *vs*. 6-OHDA alone; ^§^*p* = 0.006 *vs*. 6-OHDA + AAV-Rheb(S16H) group (*n* = 5 for each group). **e**, **f** Representative coronal SN (scale bar, 200 μm) and STR (scale bar, 1000 μm) sections stained with anti-TH at 11 weeks post-lesion. **g** Quantitative analysis showing the population of preserved TH-positive neurons in the SN. *p < 0.001 *vs*. intact controls; ^#^*p* = 0.006 *vs*. 6-OHDA alone (*n* = 3 for each group). **h** The histogram shows the optical densities of the striatal TH-positive fibers. *p < 0.001 *vs*. intact controls; ^#^*p* < 0.001 and ^##^*p* = 0.001 *vs*. 6-OHDA alone; ^§^*p* = 0.003 *vs*. 6-OHDA + AAV-Rheb(S16H) group (*n* = 3 for each group). **i** The levels of striatal dopamine, which were measured with high-performance liquid chromatography, were quantitatively expressed as a percentage of intact control. *p < 0.001 *vs*. intact controls; ^#^*p* < 0.001, ^##^*p* = 0.024 and ^###^*p* = 0.028 *vs*. 6-OHDA alone; ^§^*p* = 0.024 *vs*. 6-OHDA + AAV-Rheb(S16H) group [*n* = 4 for AAV-GFP + 6-OHDA + AAV-Rheb(S16H) group; *n* = 5 for the other groups]. One-way ANOVA with Tukey’s *post hoc* test was used in **d** and **g**–**i**

To determine the importance of sustaining the increased levels of neuronal AEG-1 in the adult nigrostriatal DA system and, consequently, the potential of AEG-1 overexpression as a therapeutic approach for PD, we examined the effects of AEG-1 overexpression following post treatment with AAV-Rheb(S16H) on the functional recovery of nigral DA neurons and induction of axonal regeneration in damaged DA neurons^9,10,12^. As shown in the experimental schematic (Fig. 5a), treatment with 6-OHDA alone induced significant reductions in the motor performance, which was measured using the open-field test (Fig. 5b, c) and rotarod test (Fig. 5d), compared to that in noninjected controls (*p < 0.001 *vs*. intact controls). Similar to the results described in our previous report^9^, Rheb(S16H) overexpression rescued the motor impairments induced by 6-OHDA neurotoxicity compared to mice injected with 6-OHDA alone (^#^*p* = 0.008 and ^##^*p* = 0.01 *vs*. 6-OHDA alone). In particular, we found that AEG-1-overexpressing mice that were injected with AAV-Rheb(S16H) following 6-OHDA injections demonstrated significant reversals of the motor impairments that were caused by 6-OHDA neurotoxicity compared with Rheb(S16H)-treated mice without virally overexpressed AEG-1 [Fig. 5b–d; ^§^*p* = 0.007 and ^§§^*p* = 0.007 for open-field test and rotarod test, respectively, *vs*. 6-OHDA + AAV-Rheb(S16H)]. Consistent with these results, the immunohistochemical staining of TH demonstrated that Rheb(S16H) overexpression following 6-OHDA administration induced axonal regeneration in damaged DA neurons (Fig. 5f, h; ^#^*p* < 0.001 *vs*. 6-OHDA alone)^9,10^, and Rheb(S16H) overexpression in the presence of increased levels of AEG-1 significantly restored the density of DA fibers in the STR compared to the effects in the absence of AEG-1 [Fig. 5f, h; ^§^*p* = 0.003 *vs*. 6-OHDA + AAV-Rheb(S16H)]. Moreover, the depleted levels of striatal dopamine following 6-OHDA administration, which were measured using HPLC, were greatly restored following Rheb(S16H) overexpression in the presence of virally overexpressed AEG-1 compared to the levels in its absence [Fig. 5i; ^§^*p* = 0.024 *vs*. 6-OHDA + AAV-Rheb(S16H)]. Similarly, the levels of the metabolites of dopamine, including DOPAC and HVA, were restored by Rheb(S16H) overexpression, and the effects were more obvious in the presence of overexpressed AEG-1 than in its absence (Supplementary Figure S6).

**Fig. 6:**
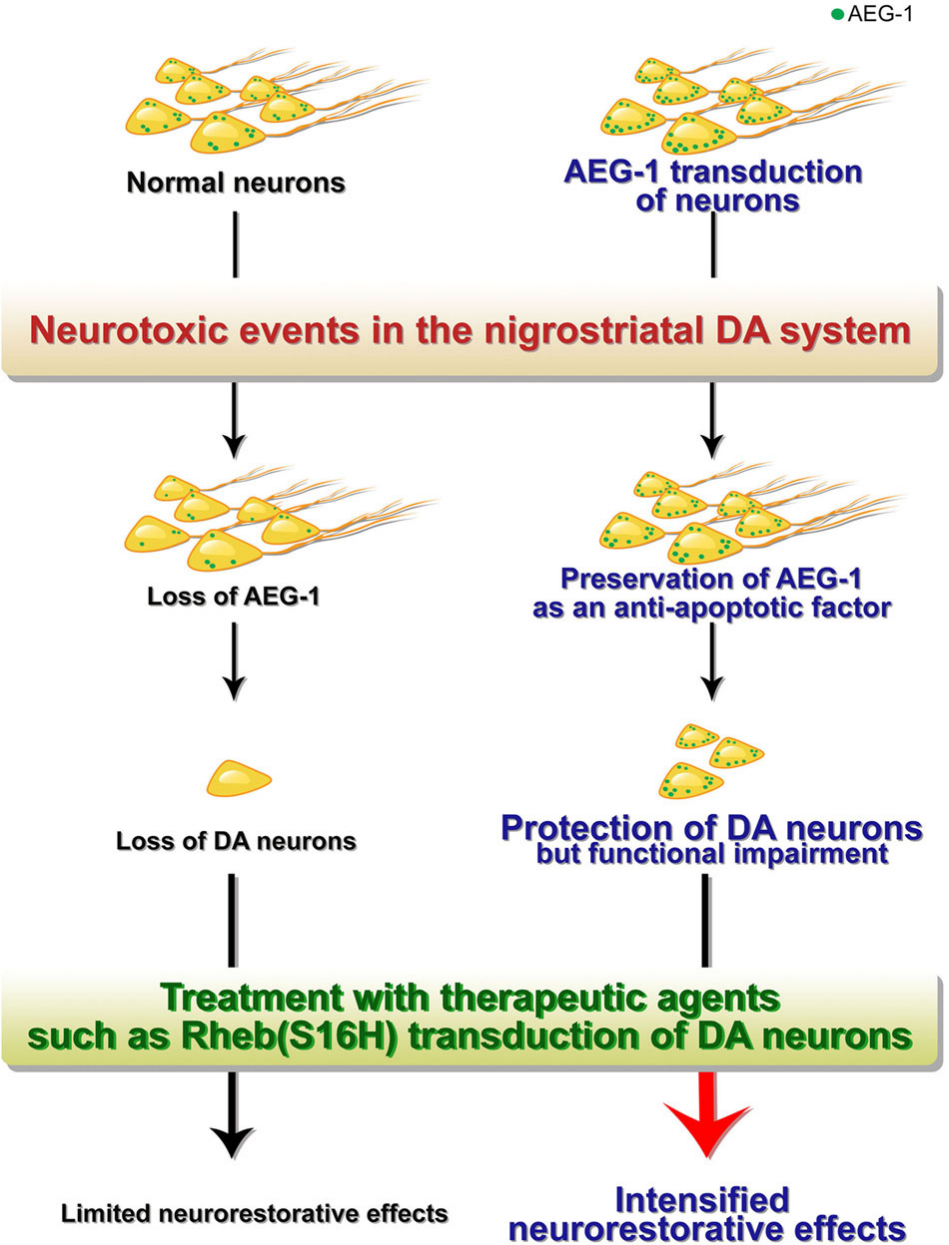
Schematic of the importance of neuronal AEG-1 preservation in the nigrostriatal DA system. Following neurotoxic events in the nigrostriatal DA system, AEG-1 transduction in DA neurons prevents the apoptotic cell death of the DA neurons (but not their functional properties). Meanwhile, decreases in the levels of neuronal AEG-1 and the consequent loss of DA neurons were observed. Moreover, AEG-1 transduction in DA neurons can intensify the neurorestorative effects by synergizing with the administration of therapeutic agents for axonal regeneration and the inhibition of abnormal activation of the autophagy–lysosomal pathway. Thus, these results suggest the importance of AEG-1 preservation in potential therapeutic strategies against PD

Rheb(S16H) administration did not affect the levels of expression of AEG-1 in the SN of the mice (Supplementary Figure S7), which suggested that the neurorestorative effects from this administration might be independent of AEG-1 expression and that its upregulation might activate the Akt/mTORC1 signaling pathway as a supplementary mechanism in the presence of AEG-1. These data suggested that AEG-1, which is significantly reduced in the SN of patients with PD, is an important endogenous factor that protects nigral DA neurons from neurotoxicity and that this protection by the anti-apoptotic effects of neuronal AEG-1 enhances the restoration of the disrupted nigrostriatal DA system (Fig. 6), as shown by the effects of Rheb(S16H) administration in the neurotoxin model of PD (Fig. 5).

## Discussion

Under physiological conditions, apoptosis is an essential homeostatic mechanism that maintains the cell population in healthy tissue and protects cells from injury^24,25^. However, aberrant apoptosis, which is one of the neurotoxic events in the adult brain, may inevitably be related to neurodegenerative diseases, such as PD and AD^6,8,26^. Moreover, consistent with the results of studies on patients with PD^6,8^, increases in the levels of apoptotic markers, such as cleaved caspase-3 and cleaved PARP-1, are observed in *in vivo* and *in vitro* models of PD^15,16^. As reported previously, the inhibition of apoptotic pathways protects DA neurons against neurotoxin treatment^7,27–30^. Thus, these results suggest that the expression and maintenance of endogenous anti-apoptotic factors are beneficial for the survival of nigral DA neurons in the adult brain.

The downregulation of AEG-1 contributes to the apoptosis of motor neurons by inhibiting the PI3K/Akt signaling pathway in *in vivo* and *in vitro* models of ALS^5^. Although the role of AEG-1 in the pathogenesis of PD was unknown, these results suggest that AEG-1 might be critical for the survival of DA neurons in the SN of patients with PD. Here we observed decreased expression of AEG-1 in damaged DA neurons in the adult brain in an immunohistochemical analysis of the SN of patients with PD and 6-OHDA-treated mice (Fig. 1b, d). However, no significant reductions in AEG-1 were observed in the hippocampus of patients with AD compared to age-matched controls (Fig. 1g). Thus, these observations suggested that the decreased levels of AEG-1 were a specific event that occurred in damaged DA neurons and AEG-1 downregulation and the loss of nigral DA neurons in PD might be clinically correlated.

Here, AAV-mediated overexpression of AEG-1 in DA neurons decreased the levels of apoptotic markers, including cleaved caspase-3 and cleaved PARP-1, following 6-OHDA administration (Fig. 3c, d)^15,16^, resulting in neuroprotection in the SN (Fig. 4b, d). The AEG-1-induced anti-apoptotic effects were confirmed by western blot analyses of the Bcl-2/Bax ratio (Supplementary Figure S1). An increase in the levels of apoptotic signaling molecules, which was similar to the results obtained in the mouse model (Fig. 3c, d), were also observed in the SN of patients with PD (Fig. 3a). These results suggested that AEG-1 plays a role as a negative regulator of apoptosis in adult DA neurons and the sustained levels of neuronal AEG-1 following neurotoxic events may confer neuroprotection to nigral DA neurons *in vivo*. Moreover, the overexpression of microglial AEG-1 in the 6-OHDA-based mouse model of PD did not confer neuroprotection (Fig. 4e, f). Therefore, consistent with the effects of reduced AEG-1 expression in motor neurons in an ALS mouse model^5^, our results showed that the sustained increased levels of neuronal AEG-1 were important for attenuating the vulnerability of nigral DA neurons in the SN of adult brain.

Despite the significant anti-apoptotic effects of AEG-1, its overexpression was not sufficient to protect the whole nigrostriatal DA projection against 6-OHDA-induced neurotoxicity (Fig. 4c, d and Supplementary Figure S2). These observations indicated limitations in the protective effects of AEG-1 against 6-OHDA-induced neurotoxicity.

One explanation for the observation that AEG-1 overexpression in DA neurons was insufficient to protect the DA system seems related to the mTORC1 pathway. The mTOR kinase, which plays a central role in the integration of responses to various environmental conditions, exists in two complexes, mTORC1 and mTORC2^10,31,32^. mTORC1 is an important mediator of Akt. mTORC2 can activate Akt, which in turn can act on mTORC1. Activation of the Akt/mTOR signaling pathway enhances the activity of cell survival pathways under various conditions, including trophic factor withdrawal, ischemic shock, and oxidative stress^9–12,32^. Moreover, recent reports have showed that the activation of neuronal mTORC1, which is a key biomolecule for neurotrophic support, by either the delivery of a specific gene or the direct administration of trophic factors induces protective effects against neurodegeneration in animal models of PD^11,33^. We recently demonstrated that the Rheb(S16H) delivery-induced activation of mTORC1 in DA neurons protects and reconstructs the damaged nigrostriatal DA projections in a mouse model of PD, which suggested that the activation of the Akt/mTORC1 signaling pathway might be a promising therapeutic strategy for the functional recovery of DA neurons^9,10^. Here, however, our results indicated that AEG-1 upregulation in nigral DA neurons did not enhance Akt/mTORC1 signaling in healthy mice (Supplementary Figure S4). Moreover, the activation of the Akt/mTORC1 signaling pathway suppressed the initiation of autophagy and prevented the aberrant accumulation of autophagic components, which might inhibit normal lysosomal degradation, such as the removal of expended macromolecules and organelles, in the nigrostriatal DA system^12,19^. Although autophagy is physiologically important for preserving cellular homeostasis and inducing protective effects, such as the suppression of apoptosis and axonal degeneration^34–37^, its aberrant activity promotes neurodegeneration in the SN of patients with PD^38^. Therefore, these observations suggest that autophagic stress that is caused by the aberrant accumulation of autophagic components might be one of the critical mechanisms that induces the loss of DA neurons in PD.

AEG-1 is known to induce autophagy, which results in the survival of cancerous cells under metabolic stress and apoptosis resistance, and these results may underlie its considerable cancer-promoting properties^39^. However, our results indicated that AEG-1 overexpression in DA neurons had no effect on the levels of LC3-II in healthy brains (Supplementary Figure S4), and on the aberrant accumulation of LC3 and p62, which are critical for ALP activation^19,22,40^ following 6-OHDA administration (Supplementary Figure S5b). Moreover, the decrease in the activity of mTORC1 following 6-OHDA neurotoxicity was not inhibited by the presence of AEG-1 in DA neurons *in vivo* (Supplementary Figure S5b). Therefore, to potentiate the beneficial effects of AEG-1 in nigral DA neurons, it may be necessary to activate the Akt/mTORC1 signaling pathway, which suppresses the aberrant accumulation of autophagic components as a supplementary protective mechanism in the presence of AEG-1 and consequently results in enhanced neuroprotection of the nigrostriatal DA projection in the adult brain.

The overexpression of Rheb(S16H), which is a constitutively active form of Rheb and activates mTORC1^9,10^, suppresses the induction of the abnormal autophagy signaling pathway by 6-OHDA treatment^12^. To further examine if AEG-1 overexpression strengthened the neuroprotective and neurorestorative effects of therapeutic agents, such as Rheb(S16H), AAV-Rheb(S16H) was injected into the SN 3 weeks post-lesion, which is when the maximum neurodegeneration is observed after 6-OHDA treatment^9,10^, in the absence or presence of virally overexpressed AEG-1 (Fig. 5a). Our results demonstrated that Rheb(S16H) upregulation in the presence of increased levels of AEG-1 induced synergistic neurorestorative effects, such as restored motor functions, in the nigrostriatal DA system disrupted by 6-OHDA neurotoxicity (Fig. 5b–i). As shown in Supplementary Figure S7, the Rheb(S16H) transduction of DA neurons did not affect the levels of AEG-1 in the SN of the mice, suggesting that the Rheb(S16H)-induced neurorestoration was independent of AEG-1 upregulation and that the AEG-1-induced neuroprotection against 6-OHDA neurotoxicity augmented the beneficial effects of Rheb(S16H) in the lesioned nigrostriatal DA system *in vivo* (Fig. 5).

In conclusion, our findings suggested that AEG-1 functioned as an anti-apoptotic factor in nigral DA neurons of the adult brain and the decrease in AEG-1 might be involved in the loss of DA neurons, which is one of the key pathological features in PD. However, the overexpression of AEG-1 in DA neurons was not sufficient to protect the whole nigrostriatal DA projection in the animal model of PD owing to its limited protective effects as it did not affect the aberrant accumulation of autophagic components and Akt/mTORC1 activity following 6-OHDA administration, which could contribute to the neurotoxic effects on the nigrostriatal DA system. To overcome this limitation of AEG-1, we further transduced the Akt/mTORC1 activator Rheb(S16H) into AEG-1-overexpressing DA neurons. Surprisingly, the synergistic effects of the two factors restored the nigrostriatal DA system that was disrupted by 6-OHDA administration, and the effects were more obvious in the presence of AEG-1 than in its absence (Figs. 5 and 6). Therefore, we concluded that AEG-1 was an important endogenous factor for protecting nigral DA neurons from aberrant apoptotic signaling pathway in the adult brain and that the maintenance of increased levels of AEG-1 in nigral DA neurons in patients with PD, in combination with therapeutic agents, such as an Akt/mTORC1 signaling activator, may be a highly promising therapeutic strategy to maximize the functional recovery of the damaged nigrostriatal DA system (Fig. 6).

## Materials and methods

### Ethics statement

All animal experiments were performed in accordance with the approved animal protocols and guidelines established by the Animal Care Committee at Kyungpook National University (Number: KNU 2016-42). Experiments involving human tissue were approved by the Bioethics Committee, Institutional Review Board Kyungpook National University Industry Foundation (IRB Number: KNU 2014-0007 and 2016-0011).

### Human brain tissue

Frozen and paraffin-fixed brain tissues were obtained from the Victorian Brain Bank Network (VBBN), supported by the Florey Institute of Neuroscience and Mental Health, The Alfred, and the Victorian Forensic Institute of Medicine, and funded by Australia’s National Health & Medical Research Council and Parkinson’s Victoria. Frozen and paraffin-fixed brain tissues were used in quantitatively analyzing the level of proteins and in observing the expression pattern of target molecule, respectively.

### Materials

Materials were purchased from the following companies: 6-OHDA (Sigma, St Louis, MO), desipramine (Sigma), l-ascorbic acid (Sigma), rabbit anti-TH (Pel-Freez, Brown Deer, WI), mouse anti-TH (R&D Systems, Minneapolis, MN), rabbit anti-Iba1 (Wako Pure Chemical Industries, Osaka, Japan), rabbit anti-GFAP (Millipore, Billerica, MA), rabbit anti-AEG-1 (Invitrogen, Camarillo, CA), rabbit anti-GFP (Millipore), mouse anti-HA (Cell Signaling, Beverly, MA), rabbit anti-HA (Cell Signaling), rabbit anti-FLAG (Sigma), rabbit anti-caspase-3 (Cell Signaling), rabbit anti-cleaved caspase-3 (Cell Signaling), rabbit anti-PARP-1 (Cell Signaling), rabbit anti-cleaved PARP-1 (Cell Signaling), rabbit anti-LC3B (Cell Signaling), rabbit anti-4E-BP1 (Cell Signaling), rabbit anti-p-4E-BP1 (Cell Signaling), mouse anti-NeuN (Millipore), rabbit anti-Akt (Cell Signaling), rabbit anti-p-Akt (Cell Signaling), rabbit anti-β-actin (Cell Signaling), rabbit anti-α-tubulin (Cell Signaling), mouse anti-Bcl-2 (Santa Cruz Biotechnology, Santa Cruz, CA), mouse anti-Bax (Santa Cruz Biotechnology), rabbit anti-p62/SQSTM1 (Sigma), biotinylated anti-rabbit IgG (Vector laboratories, Burlingame, CA), Texas Red-conjugated anti-rabbit/mouse IgG (Vector Laboratories), fluorescein (FITC)-conjugated anti-mouse IgG (Vector Laboratories), FITC-conjugated anti-rabbit IgG (Jackson ImmunoResearch Laboratories, Bar Harbor, ME), horseradish peroxidase (HRP)-conjugated anti-rabbit IgG (Enzo Life Sciences, Farmingdale, NY) and HRP-conjugated anti-mouse IgG (Thermo Fisher Scientific, Rockford, IL).

### Production of viral vectors

The two types of viral vectors used in the present study were AAV serotype 1 and Ad serotype 5. Viral vectors were produced as described previously, with some modifications^10,17,41,42^. Ad viral vectors were supplied by S.G. Lee. Briefly, for the production of AAV viral vectors carrying AEG-1 with a HA-encoding sequence at the 3ʹ-end (AEG-1-HA), AEG-1 cDNA tagged with HA was amplified from the mammalian expression plasmid of AEG-1 (pcDNA3.1-AEG-1-HA), as previously described^17,42^. AEG-1-HA obtained from pcDNA3.1-AEG-1-HA was cloned into an AAV packaging construct that utilizes the chicken β-actin promotor and contains a 3′ WPRE^10^. Constitutively activated Rheb was also cloned into the same AAV packaging construct^10^. All nucleotide sequences in the AAV packaging construct were confirmed before viral vector production. AAVs were produced at the University of North Carolina Vector Core. The genomic titer of AAV-AEG-1 and AAV-Rheb(S16H) were 9.4 × 10^12^ viral genomes/ml and 3.6 × 10^12^ viral genomes/ml, respectively. Enhanced GFP, used as a control, was subcloned into the same AAV viral backbone, and viral stock was produced at a titer of 2.0 × 10^12^ viral genomes/ml. Genomic titers of both Ad-AEG-1 and Ad-null viral stocks were 2.0 × 10^9^ infectious unit/ml.

### Intranigral AAV and Ad injection

Adult (8- to 10-week-old) male C57BL/6 mice were obtained from Daehan Biolink (Eumseong, Korea). As previously described, with some modifications^9,10^, mice were anesthetized with chloral hydrate solution and placed in a stereotaxic frame (Kopf Instruments, Tujunga, CA) with a mouse adapter. Each mouse received a unilateral injection of AAV or Ad into the right SN (AP: −0.35 cm, ML: −0.11 cm, DV: −0.37 cm, relative to bregma) using a 30-gauge Hamilton syringe attached to an automated microinjector^43^. Viral vector suspension in a volume of 2.0 μl was injected at a rate of 0.1 μl/min over 20 min. After injection, the needle was left in place for an additional 5 min before being slowly retracted.

### Intrastriatal 6-OHDA injection

The intrastriatal 6-OHDA model was induced as previously described^9,10^. Mice were intraperitoneally injected with desipramine (25 mg/kg in 0.9% NaCl), and then anesthetized with chloral hydrate. Anesthetized mice were placed in a stereotaxic frame, and a solution of 6-OHDA (5 mg/ml in 0.9% NaCl/0.02% ascorbic acid), with a final volume of 3.0 μl was injected by Hamilton syringe at a rate of 0.5 μl/min. The injection was performed into the right STR at coordinates (AP: +0.09 cm; ML: −0.22 cm; DV: −0.25 cm, relative to bregma)^43^. The needle was withdrawn slowly after 5 min. Animals were sacrificed and analyzed at the indicated time points for each experiment^9,10^.

### Behavioral tests

#### Open-field test

The open-field test was performed as described previously, with some modifications^13^. Briefly, 11 weeks after the 6-OHDA injection, mice were placed individually in the corner of a test chamber (40 × 40 × 40 cm) enclosed with white acrylic walls. After a 1 min adaptation period, animal behaviors such as the total distance traveled (in cm) and velocity (in cm/sec) were recorded for 5 min using a video camera. The change in locomotor activity was analyzed offline by video-tracking software (SMART, Panlab, Barcelona, Spain). The test chamber was cleaned between trials with 70% ethyl alcohol. To minimize stress levels, tests were performed under conditions of low illumination.

#### Rotarod test

The rotarod test was performed at 11 weeks post-lesion, using a previously described procedure with some modifications^13^. Before 6-OHDA treatment, all mice were pre-trained on the rotarod apparatus (3 cm rod diameter; Scitech Inc., Seoul, Korea) at 10 revolutions per min (rpm) for 10 min, and the training was performed for 3 consecutive days. Eleven weeks after the 6-OHDA injection, performance on the rod was evaluated at a constant acceleration rate of 4–40 rpm in 300 sec. Two consecutive trials were performed at 60 min intervals.

### Immunohistochemical staining

Postmortem brain tissues were processed for immunohistochemistry as described previously^44^. Briefly, the human SN sections were deparaffinized and subjected to citrate-based antigen retrieval, and then washed in cold PBS and blocked with blocking solution. The sections were incubated with primary antibodies against AEG-1 (1:500) at 4 °C overnight, and then incubated with biotinylated secondary antibodies for 1 h at room temperature, followed by addition of the avidin-biotin reagent (Vectastain ABC kit, Vector Laboratories) for 1 h at room temperature. The SN sections were visualized using a 3,3′-diaminobenzidine (DAB; Sigma) peroxidase substrate solution [0.05% DAB, 0.05% Cobalt Chloride (Sigma), 0.05% Nickel Ammonium Sulfate (Sigma) and 0.015% H_2_O_2_ in PBS, pH 7.2]. Each section was covered with a thin glass coverslip and analyzed under a bright-field microscope (Carl Zeiss, Oberkochen, Germany).

As previously described^9,10,44^, mice were transcardially perfused and fixed, and the brains were dissected out, frozen, and cut into 30-μm-thick coronal sections using a cryostat microtome (Thermo Fisher Scientific). Briefly, the brain sections were washed in PBS and blocked with blocking buffer, and then incubated at 4 °C for 2 days with the following primary antibodies: rabbit anti-TH (1:2000), rabbit anti-AEG-1 (1:500), rabbit anti-Iba1 (1:2000), rabbit anti-GFAP (1:1000), mouse anti-HA (1:1000), rabbit anti-cleaved caspase-3 (1:400), rabbit anti-cleaved PARP-1 (1:400) and mouse anti-TH (1:500). After incubation, the brain sections were incubated with biotinylated secondary antibodies, followed by addition of the avidin-biotin reagent (Vectastain ABC kit) for 1 h at room temperature, or incubated with fluorescence-conjugated secondary antibodies for 1 h. The signal following treatment with avidin-biotin reagent was detected by incubating the sections in 0.5 mg/ml DAB (Sigma) in 0.1 M PB containing 0.003% H_2_O_2_. The sections incubated with fluorescence-conjugated secondary antibodies were washed in 0.1 M PBS. The stained sections were mounted on gelatin-coated slides and analyzed under a light or fluorescence microscope (Carl Zeiss).

### Western blot analysis

Animal and human tissue samples were prepared for western blot analysis as previously described^10,44^. Briefly, animal SN tissues were removed and sliced using a brain matrix (Roboz Surgical Instrument Co., Gaithersburg, MD). Animal or human tissue samples were homogenized and centrifuged at 4 °C for 20 min at 14,000 × *g*; the supernatant was transferred to a fresh tube and the concentration was determined using a bicinchoninic acid assay (BCA) kit (Bio-Rad Laboratories, Hercules, CA). Aliquots containing 50 μg of protein were electrophoresed on a sodium dodecyl sulfate (SDS)/polyacrylamide gel (Bio-Rad Laboratories) and transferred to polyvinylidene difluoride (PVDF) membranes (Millipore, Billerica, MA) using an electrophoretic transfer system (Bio-Rad Laboratories) The membranes were incubated overnight at 4 °C with the following primary antibodies: rabbit anti-TH (1:2000), rabbit anti-AEG-1 (1:1000), mouse anti-NeuN (1:500), rabbit anti-GFP (1:500), rabbit anti-HA (1:1000), rabbit anti-FLAG (1:3000), rabbit anti-caspase-3 (1:1000), rabbit anti-cleaved caspase-3 (1:1000), rabbit anti-PARP-1 (1:1000), rabbit anti-cleaved PARP-1 (1:1000), mouse anti-Bcl-2 (1:1000), mouse anti-Bax (1:1000), rabbit anti-LC3B (1:1000), rabbit anti-p62/SQSTM1 (1:1000), rabbit anti-Akt (1:1000), rabbit anti-p-Akt (1:2000), rabbit anti-4E-BP1 (1:1000), and rabbit anti-p-4E-BP1 (1:1000). Subsequently, the membranes were incubated with secondary antibodies for 1 h at room temperature, and the bands were finally detected using Western-blot detection reagents (Thermo Fisher Scientific, Rockford, IL). For quantitative analyses, the density of each band was measured using a Computer Imaging Device and accompanying software (Fuji Film, Tokyo, Japan), and the levels were quantitatively expressed as the density normalized to the housekeeping protein band for each sample.

### Stereological estimation

As previously described^10,44^, the total number of TH-positive neurons was counted in the various animal groups using the optical fractionator method. Counting of TH-positive neurons in the SN was performed on a bright-field microscope (Olympus Optical, BX51, Tokyo, Japan) using Stereo Investigator software (MBF Bioscience, Williston, VT). This unbiased stereological method of cell counting is not affected by either the reference volume (SN pars compacta) or the size of the counted elements (neurons).

### Quantitative determination of striatal TH immunoperoxidase staining

Densitometric analysis of the mouse STR was carried out as previously described^10,44^. Briefly, an average of 6 coronal sections of the STR that gathered according to the bregma of the brain atlas^43^ was imaged at a ×1.25 magnification. The density of striatal TH-positive fibers was measured using the Science Lab 2001 Image Gauge (Fujifilm, Tokyo, Japan). To control for variations in background illumination, the density of the corpus callosum was subtracted from the density of STR for each section. The density in both the contralateral and ipsilateral sides was expressed by comparing with the average density of TH-positive fiber innervating the contralateral side.

### Measurement of dopamine and its metabolites in the STR

As previously described^44^, the levels of striatal dopamine and its metabolites were measured by high performance liquid chromatography (HPLC, 1260 Infinity system, Agilent Technologies, Santa Clara, CA) using an ESA Coulochem III electrochemical detector. Briefly, the brain tissues were homogenized and centrifuged, and the supernatants were injected using an autosampler at 4 °C (Waters 717 plus autosampler) and eluted through a μ Sunfire C18 column (4.6 × 100 mm × 5 μm; Waters Corporation, Milford, MA) with a mobile phase of MDTM/acetonitrile (90:10). The peaks of dopamine and its metabolites were analyzed and integrated using a ChemStation software (Agilent Technologies, Santa Clara, CA), and all samples were normalized for protein content as spectrophotometrically determined using the Pierce BCA protein assay kit (Thermo Scientific, Waltham, MA).

### Statistical analysis

Differences between two groups were analyzed using *t*-test. Multiple comparisons between groups were performed using one-way analysis of variance (ANOVA) followed by Tukey’s *post hoc* test. Behavioral test was analyzed using one-way ANOVA and *t*-tests. All values represent the mean ± standard error of the mean (SEM), and statistical analyses were performed using SigmaStat software (Systat Software, San Leandro, CA).

## Electronic supplementary material

Supplementary Materials
