# Supplementary material for: Upregulation of neuronal astrocyte elevated gene-1 protects nigral dopaminergic neurons in vivo
Source: Cell Death Dis. 2018 Apr 18;9(5):449. doi: 10.1038/s41419-018-0491-3 (PMC5906475; doi:10.1038/s41419-018-0491-3)
Supplement: Supplementary file 1 — Supplementary Materials [file 41419_2018_491_MOESM1_ESM.docx]

**Supplementary Materials for**

**Upregulation of neuronal astrocyte elevated gene-1 protects nigral dopaminergic neurons *in vivo***

Leem, E. *et al*.

Supplementary Figures 1-7 and Figure Legends

**
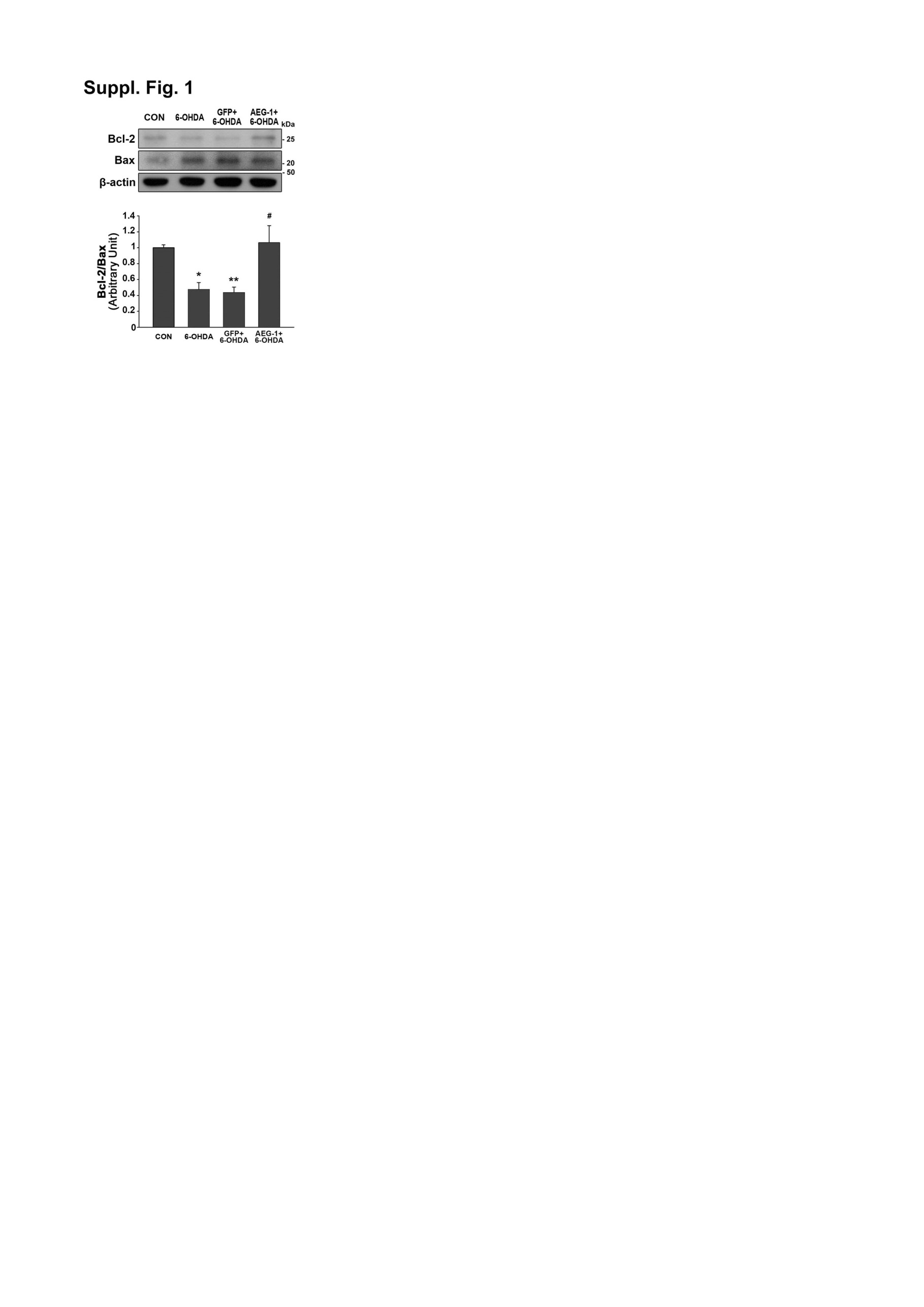
**

**Supplementary Figure 1**

AEG-1 transduction of DA neurons induces a significant increase in the ratio of Bcl-2/Bax against 6-OHDA-induced neurotoxicity.

Similar to the effect of AEG-1 overexpression on levels of pro-apoptotic signaling molecules upon 6-OHDA treatment (Figure 3), western blot analysis shows that neuronal AEG-1 overexpression induces a significant increase in the Bcl-2/Bax ratio in the SN at 2 days after 6-OHDA-induced neurotoxicity. All values represent the mean ± SEM. ^*^*p* = 0.045 and ^**^*p* = 0.031, significantly different from CON; ^#^*p* = 0.024, significantly different from 6-OHDA alone (one-way ANOVA with Tukey’s *post-hoc* test; n = 4 for each group).


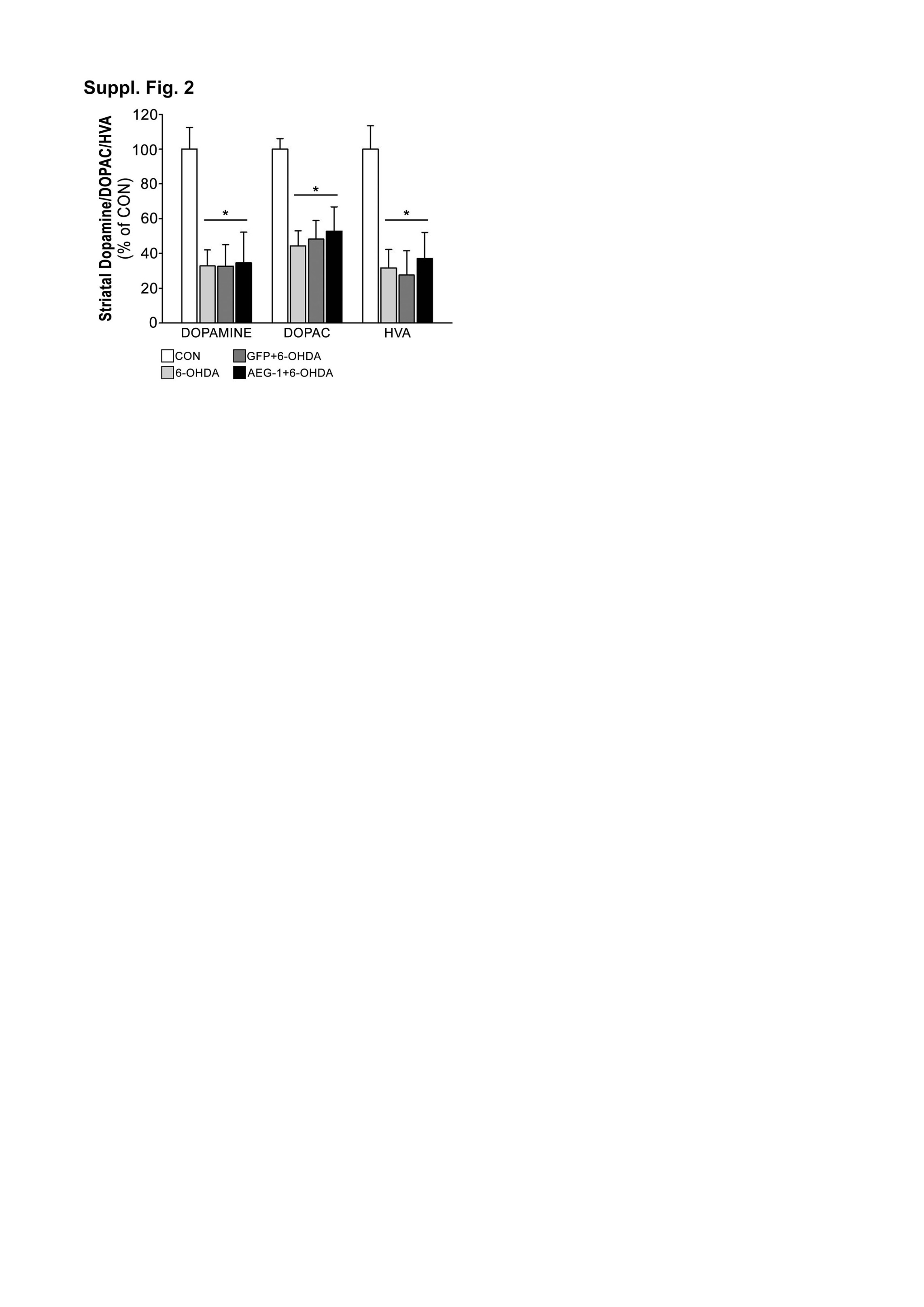


**Supplementary Figure 2**

HPLC analysis of striatal dopamine and its metabolites, DOPAC and HVA.

AEG-1 transduction of DA neurons does not ameliorate the loss of striatal DA and its metabolites following 6-OHDA-induced neurotoxicity at 7 days post-lesion. Values are expressed as a percentage of the value for the contralateral controls for each sample. ^*^*p* < 0.05 *vs*. CON (one-way ANOVA with Tukey’s *post-hoc* test; n = 4 for each group). All values represent the mean ± SEM.


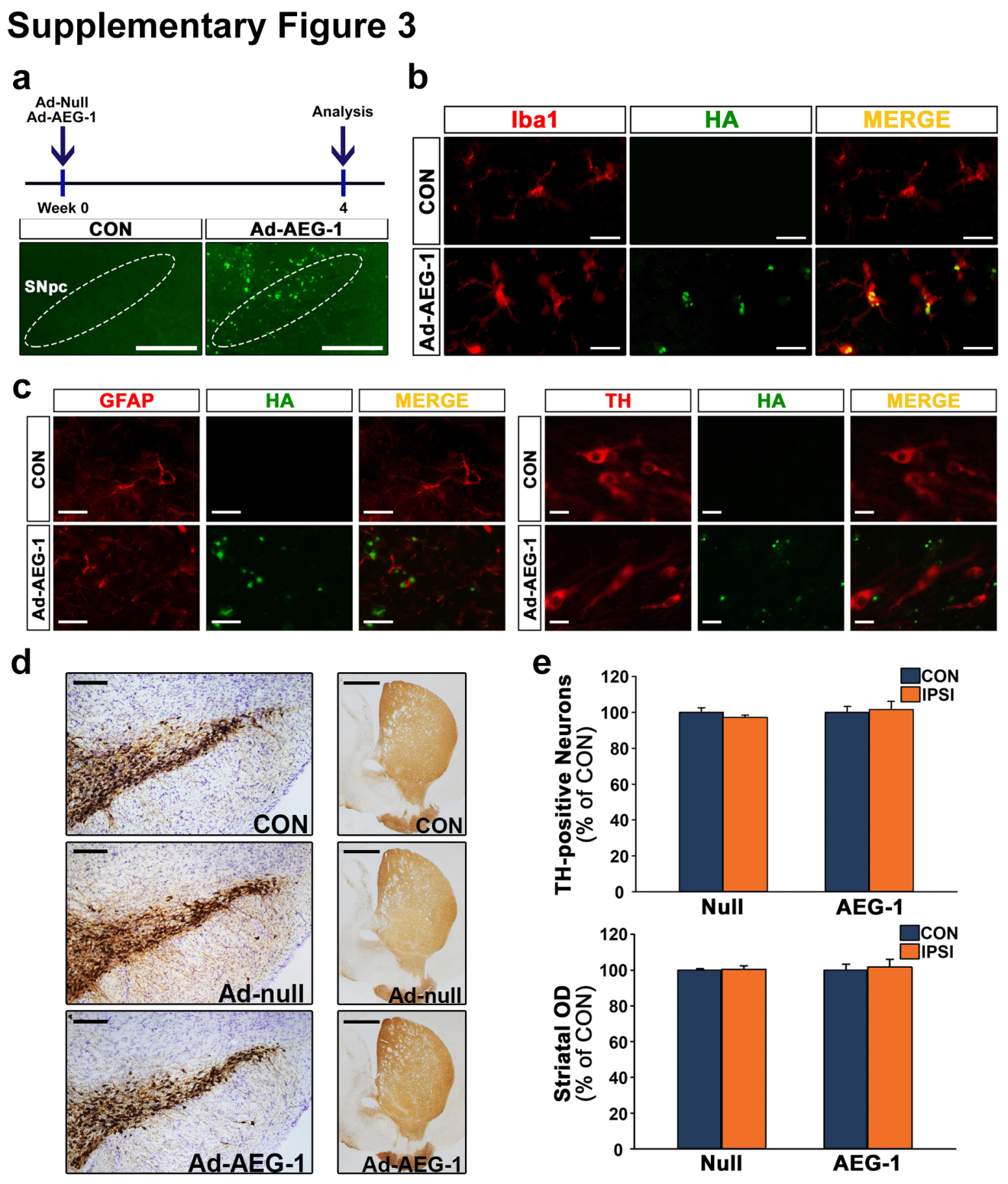


**Supplementary Figure 3**

Ad-AEG-1 transduction of microglia in the SN of healthy brain *in vivo.*

(**a**) Experimental scheme and expression of HA (green) following Ad-AEG-1 administration in the mouse SN. Inside areas of the dotted elliptical shape indicate the SNpc. Scale bar, 200 μm. (**b**) Immunofluorescence double-labeling for Iba1 (red) and HA (green) shows that transgene expression is co-localized with Iba1-positive microglia in the SN. Scale bar, 20 μm. (**c**) There is no HA expression within GFAP-positive astrocytes and TH-positive DA neurons in the Ad-AEG-1-treated SN. Scale bar, 20 μm. (**d**) Representative coronal sections indicate that transduction of Ad viral constructs, used for neuroprotective effects (Figure 4), into microglia does not induce cytotoxic effects in the nigrostriatal DA system of healthy brain. Scale bars, 200 μm for SN and 1000 μm for STR. (**e**) Quantification of the number of TH-positive neurons and striatal optical density of TH-positive fibers after injection of Ad viral constructs (one-way ANOVA with Tukey’s *post-hoc* test; n = 4 for each group). All values represent the mean ± SEM.

**
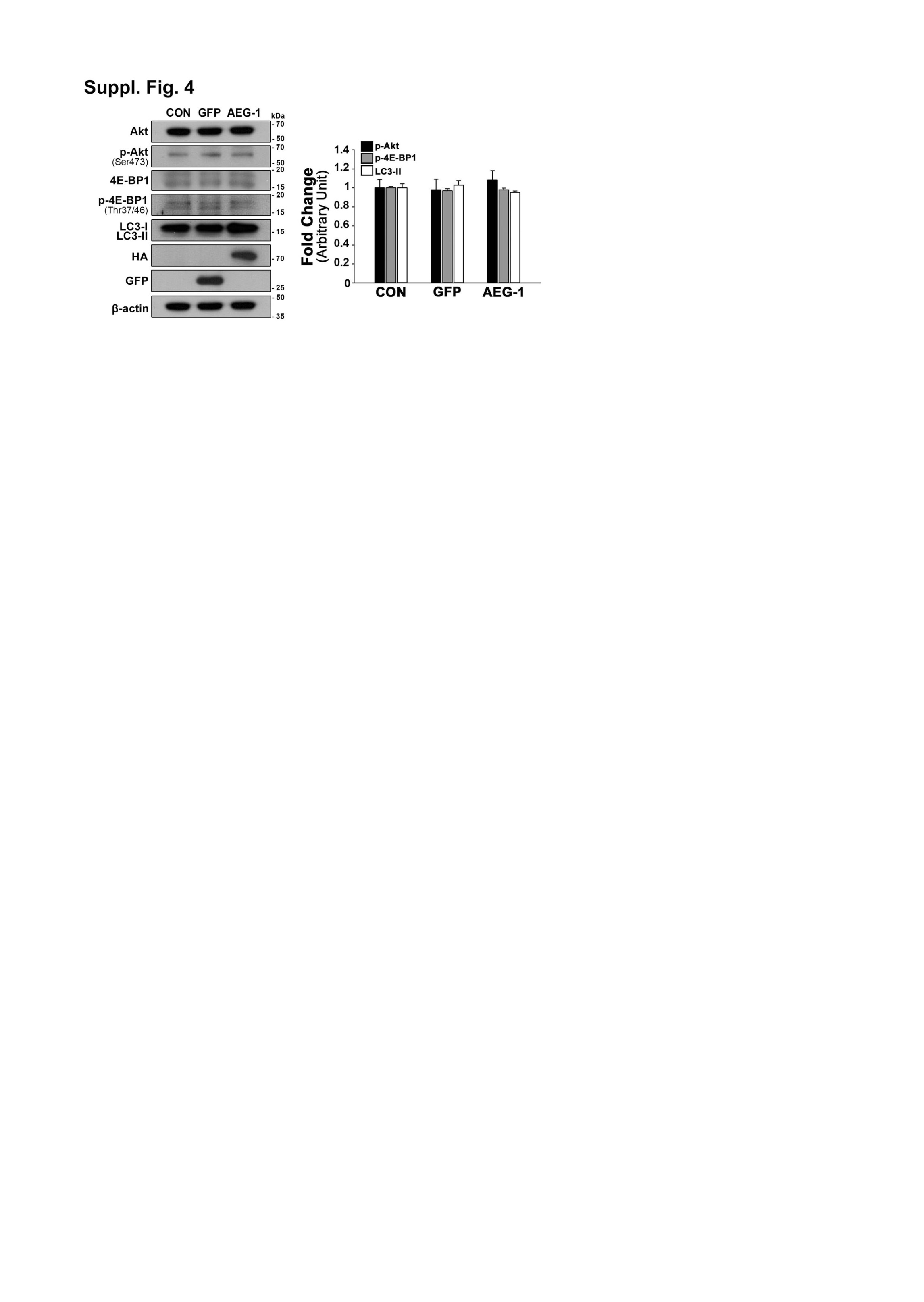
**

**Supplementary Figure 4**

Effects of AEG-1 overexpression in DA neurons on the levels of p-Akt, p-4E-BP1 and LC3 in the SN of healthy brain.

Western blot analysis shows that transduction of DA neurons with AEG-1 does not induce a significant increase in the levels of p-Akt, p-4E-BP1 and LC3-II in the SN, compared to non-injected controls 4 weeks after nigral injection of AAV-AEG-1 or AAV-GFP constructs (one-way ANOVA with Tukey’s *post-hoc* test; n = 4 for each group). All values represent the mean ± SEM.

**
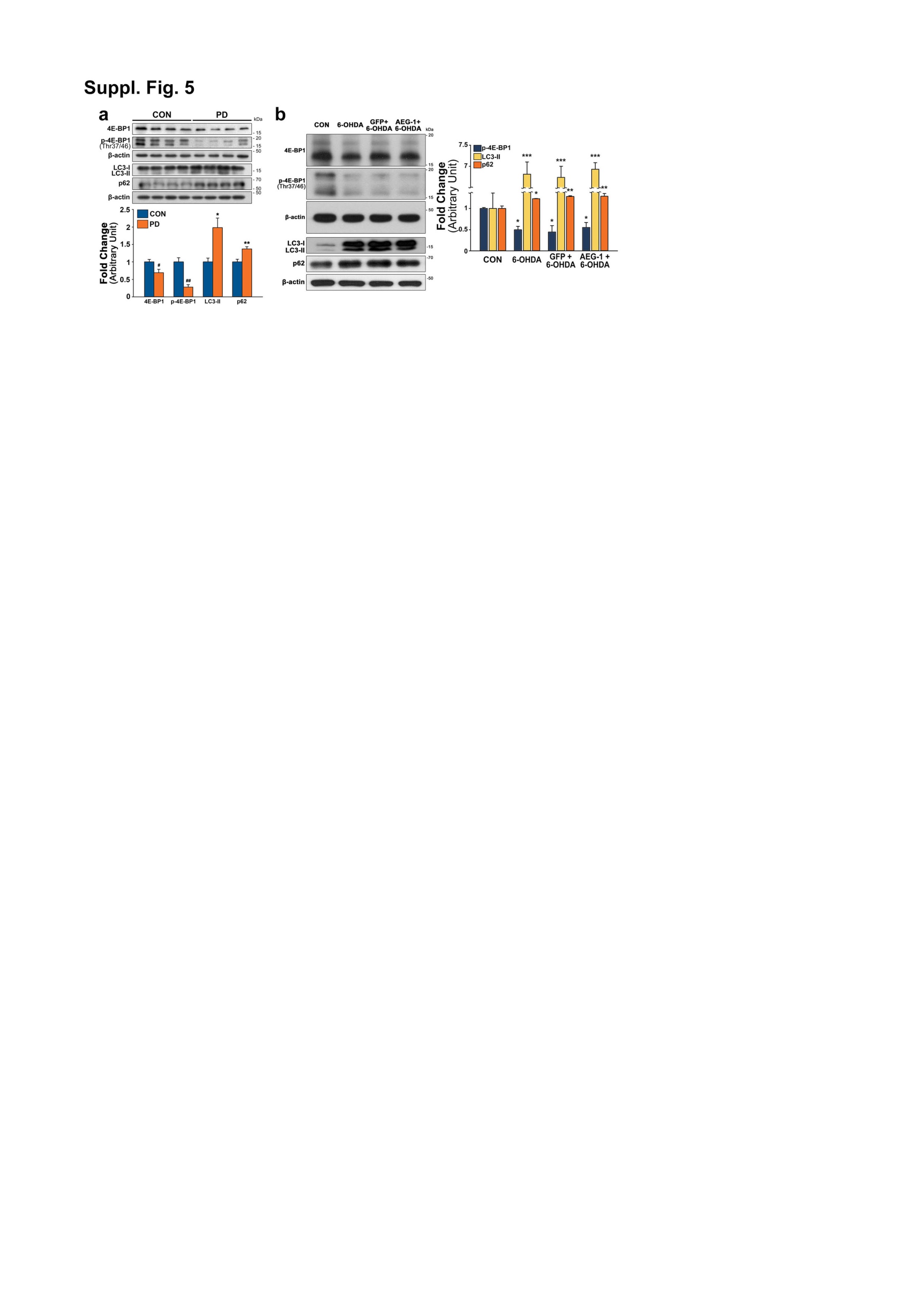
**

**Supplementary Figure 5**

Aberrant accumulation of autophagic components in the SN of PD patients and 6-OHDA-lesioned mice.

(**a**) Western blot analysis shows a significant decrease in the levels of 4E-BP1 and p-4E-BP1 and a significant increase in the levels of LC3-II and p62 in the SN of PD patients compared with age-matched controls (CON). ^#^*p* = 0.042 for 4E-BP1, ^##^*p* = 0.002 for p-4E-BP1, ^*^*p* = 0.016 for LC3-II, and ^**^*p* = 0.010 for p62 significantly different from CON (*t*-test; n = 4 for each group). All values represent the mean ± SEM. (**b**) Western blot analysis for p-4E-BP1, P62, and LC3-II shows the occurrence of autophagic stress by the aberrant accumulation of autophagic components in the 6-OHDA-treated SN. However, no significant change in the levels of those was observed in the presence of AEG-1. ^*^*p* < 0.05, ^**^*p <* 0.01, and ^***^*p* < 0.001 *vs*. CON (one-way ANOVA with Tukey’s *post-hoc* test; n = 4 for each group). All values represent the mean ± SEM.

**
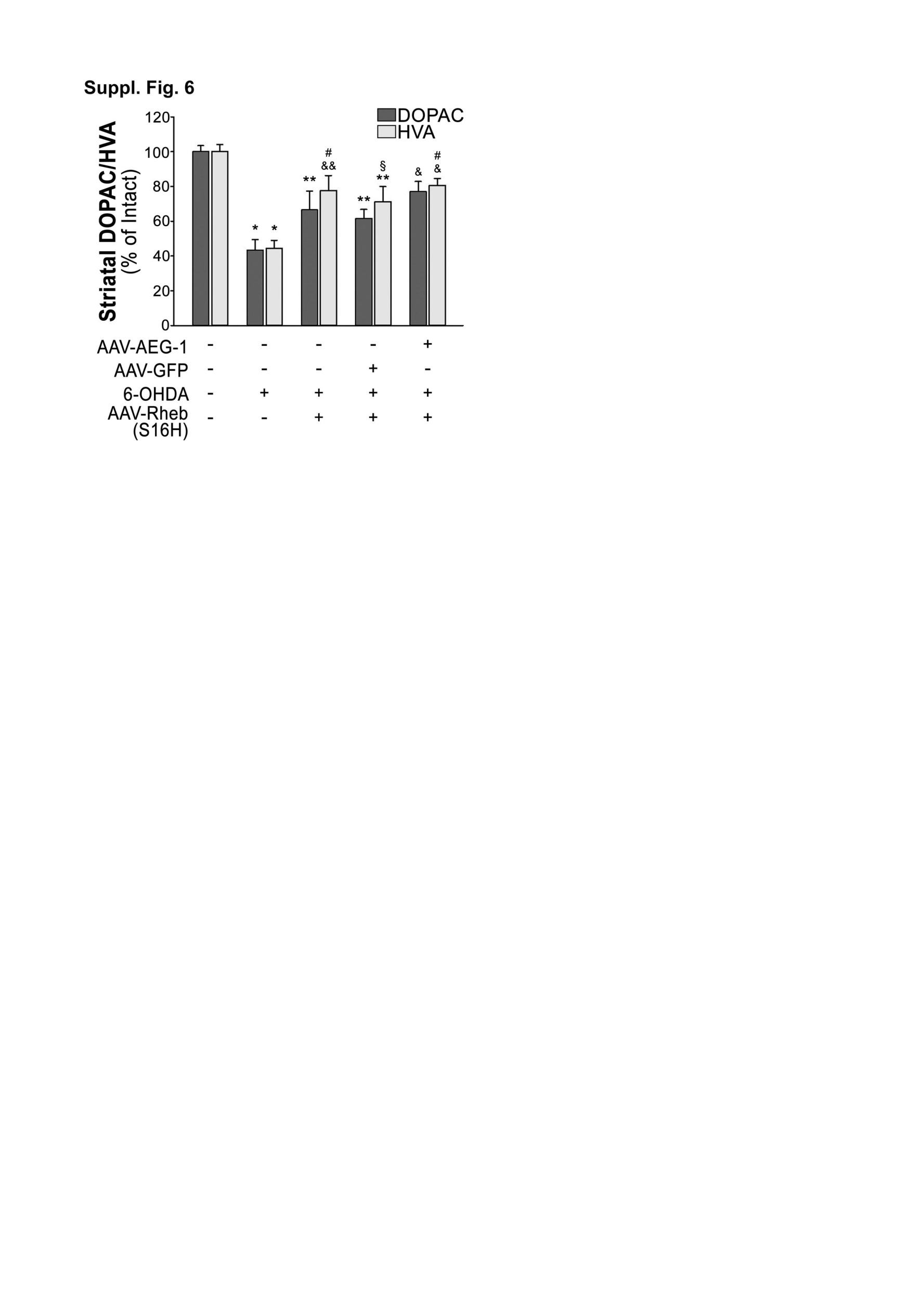
**

**Supplementary Figure 6**

HPLC analysis of striatal DOPAC and HVA.

The level of striatal DOPAC and HVA was measured by HPLC following 6-OHDA-induced neurotoxicity at 11 weeks post-lesion. The level was quantitatively expressed as a percentage of intact control. ^*^*p* < 0.01 and ^**^*p* < 0.05 *vs*. non-injected control (one-way ANOVA with Tukey’s *post-hoc* test); ^&^*p* = 0.010 and ^&&^*p* = 0.48 *vs*. non-injected control (*t*-test); ^#^*p* < 0.01 *vs*. 6-OHDA alone (one-way ANOVA with Tukey’s *post-hoc* test); ^§^*p* = 0.024 *vs*. 6-OHDA alone (*t*-test); [n = 4 for AAV-GFP + 6-OHDA + AAV-Rheb(S16H) group; n = 5 for the other groups]. All values represent the mean ± SEM.

**
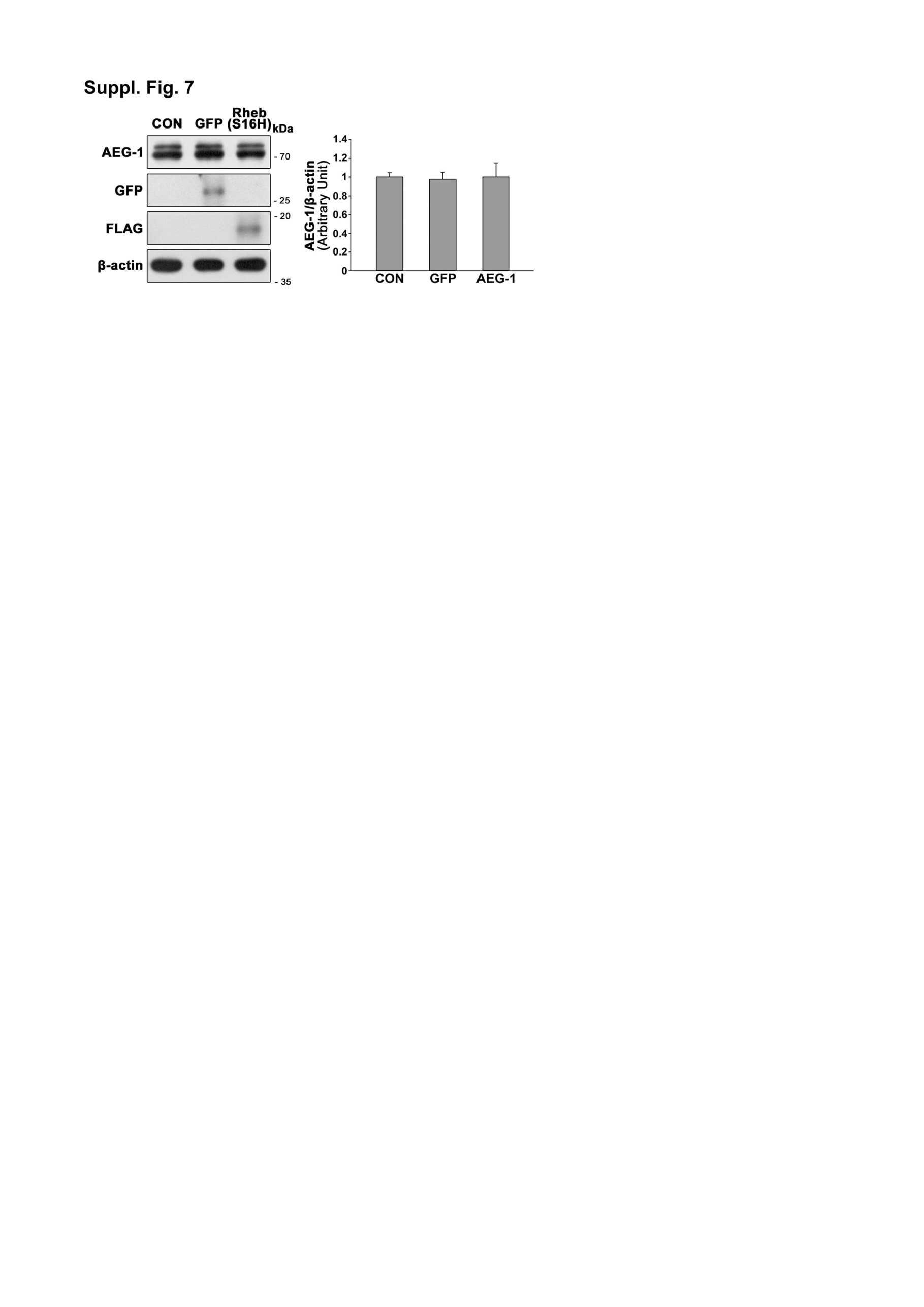
**

**Supplementary Figure 7**

No change in the level of AEG-1 following Rheb(S16H) trandcution of DA neurons.

Western blot analysis shows that there is no significant change in the level of AEG-1 following Rheb(S16H) transduction of DA neurons *in vivo* (one-way ANOVA with Tukey’s *post-hoc* test; n = 4 for each group).
